# Exploring the Effect of Enzyme and Protein-Containing Toothpaste on Gum Health: A Systematic Review

**DOI:** 10.3390/microorganisms13051158

**Published:** 2025-05-20

**Authors:** Silvia D’Agostino, Marco Dolci

**Affiliations:** Department of Medical, Oral and Biotechnological Sciences, University G. d’Annunzio, 66100 Chieti, Italy; marco.dolci@unich.it

**Keywords:** enzymes, lactoferrin, lactoperoxidase, lysozyme, toothpaste, gingivitis, microbiota

## Abstract

This systematic review critically evaluates the efficacy of enzyme- and protein-containing toothpastes in augmenting saliva’s inherent protective mechanisms. Adhering to PRISMA guidelines and having been registered under PROSPERO (CRD42024558854), a comprehensive literature search was conducted across PubMed, Web of Science, and Scopus, employing a PICO-structured query with the MeSH terms “enzymes”, “proteins”, and “toothpaste”. The inclusion criteria were restricted to in vivo human studies, published in English within the last 10 years, assessing the gingival effects of these toothpastes. Risk of bias was assessed using the Cochrane RoB 2.0 for randomized controlled trials (RCTs) and ROBINS-I for non-randomized controlled trials (N-RCTs). From an initial pool of 62 articles, three studies met the inclusion criteria: two RCTs exhibiting low to medium risk of bias and one N-RCT with low risk of bias. The analysis suggests that enzyme- and protein-enriched toothpastes may contribute to improved gingival health following 12 weeks and 12 months of use. However, the hypothesized effect against extrinsic black stains was not substantiated within the selected studies. These findings, while promising, are constrained by the limited number of included studies, necessitating further investigations to validate these observations and explore the broader implications of enzyme- and protein-based oral care formulations.

## 1. Introduction

The human oral cavity represents a complex ecological niche, hosting a diverse and dynamic microbial community integral to the maintenance of oral health. This oral microbiome comprises a consortium of microorganisms, including bacteria, fungi, viruses, and archaea. Bacteria constitute the predominant component, with over 700 distinct species identified [1]. In a state of physiological homeostasis, the oral microbiome exhibits a dynamic equilibrium, characterized by a relative abundance of commensal species that suppress the proliferation of pathogenic organisms. However, this delicate microbial balance is susceptible to perturbation by a multitude of factors, including suboptimal oral hygiene practices, host genetic predisposition, and environmental exposures. Such disruptions can precipitate dysbiosis, a pathological state characterized by a shift in the microbial community structure towards a predominance of pathogenic species [2].

In individuals with gingivitis, a higher abundance of proteolytic and often obligate anaerobic species was found [3]. Saliva contributes to shaping the composition and function of the oral microbiome. Its protective properties stem from its components, including enzymes, proteins, and antimicrobial peptides [4].

The salivary lactoperoxidase system (LPO system) catalyzes the oxidation of saliva thiocyanate ion to hypothiocyanite, a potent antimicrobial agent [5]. The hypothiocyanite produced by the LPO system demonstrates a broad-spectrum antibacterial activity against a diverse array of bacterial species, including those implicated in dental caries and periodontal diseases [6,7].

In addition to the LPO system, lysozyme breaks down bacterial cell walls, causing lysis and reducing pathogen activity. Lactoferrin binds iron, limiting bacterial growth, and directly kills certain oral pathogens. Secretory IgA (sIgA) prevents bacterial adhesion, neutralizes toxins, and helps maintain microbial balance [8].

An interesting study by Soares RV et al. [9] stated that salivary micelles have a distinct protein composition compared to whole saliva, containing a subset of proteins known to be important for the innate immune system. These proteins include high-molecular-weight proteins (MG2 and secretory IgA), intermediate-molecular-weight proteins (lactoferrin, amylase, and glycosylated proline-rich protein), and low-molecular-weight proteins (lysozyme).

Given the importance of saliva in maintaining oral health, toothpastes with enzymes and proteins have been developed to augment the natural salivary defenses [1]. These toothpastes typically contain enzymes such as amyloglucosidase, glucose oxidase, and lactoperoxidase, as well as proteins like lactoferrin and lysozyme, mimicking the enzyme and protein composition naturally present in saliva. Amyloglucosidase and glucose oxidase have been shown to work synergistically to increase the production of hydrogen peroxide in saliva, thereby enhancing the activity of the lactoperoxidase system [10].

A randomized clinical trial by Adams S.E. et al. [11] showed that a toothpaste containing enzymes and proteins led to a significant increase in 12 taxa associated with gingival health, including *Neisseria* spp. bacteria, and a significant decrease in 10 taxa associated with periodontal diseases, including *Treponema* spp. bacteria, over a 14-week period. These shifts in oral microbiome composition were associated with improvements in gingival health, as evidenced by reduced gingival inflammation, gingival bleeding, and plaque accumulation.

Moreover, toothpastes containing enzymes and proteins have been shown to enhance the antibacterial activity of saliva. For example, an in vitro study reported that treatment with a toothpaste containing enzymes and proteins increased hypothiocyanite levels in saliva, suggesting an enhanced activity of the lactoperoxidase system. Furthermore, the study demonstrated that the enzymes and proteins in the toothpaste adversely affected the bacterial membrane integrity in *Streptococcus mutans* and *Fusobacterium nucleatum*, two bacteria commonly associated with caries and periodontal diseases [12].

The role of oral hygiene regimens in the diversity of the human oral microbiome is well investigated in the literature. The subgingival plaque analysis conducted by Min K. et al. [13] highlighted the potential significance of mechanical flossing in maintaining oral health. Through viable bacterial enumeration utilizing vPCR, the study demonstrated that flossing can synergistically enhance the efficacy of mouth rinsing in reducing the total bacterial load and the abundance of *F. nucleatum* below the gingival margin. Moreover, flossing exhibited a selective inhibitory effect on *Porphyromonas gingivalis*. These findings were corroborated by clinical assessments of bleeding and inflammation, further emphasizing the role of mechanical flossing in controlling subgingival plaque in conjunction with mouth rinsing.

The aim of the present review was to assess whether toothpastes containing enzymes and proteins can supplement the natural oral defenses, promoting a balanced oral microbiome and contributing to the management of gum health.

## 2. Materials and Methods

A systematic review was conducted using the Preferred Reporting Items for Systematic Reviews and Meta-Analyses (PRISMA) guidelines for systematic reviews and meta-analysis [14] and registered in PROSPERO—the International Prospective Register of Systematic Reviews—with the ID code CRD42024558854.

### 2.1. Literature Search

The objective of the literature search was to define pertinent studies analyzing the effect of toothpastes containing enzymes and proteins on gum health in the last ten years. An exhaustive search of PubMed, Web Of Science, and Scopus, using the Patient/Population/Problem, Intervention, Comparison, and Outcome (PICO) format, was conducted.

- Population: humans of all ages;
- Intervention: toothpaste with enzymes and proteins tested in humans;
- Comparator: effect on gingivitis;
- Outcomes: possible effects of a toothpaste with enzymes and proteins on gingivitis and gum health.

The following MeSH (Medical Subject Headings) terms were used: enzymes; AND proteins; AND toothpaste.

### 2.2. Eligibility Criteria

The inclusion criteria were as follows: all in vivo studies on humans analyzing the gum effects of a toothpaste containing enzymes and proteins, in the English language, published in the last 10 years.

The following served as the exclusion criteria: research on periodontitis, peri-implantitis, or toothpaste with other technologies; papers about oral probiotics and non-oral environments; systematic reviews; metanalyses; editorials; abstracts; book chapters; papers not in English.

### 2.3. Data Extraction

Studies were autonomously evaluated by two reviewers (S.D., M.D.), and a matrix of relevant data was provided. If the reviewers disagreed, consensus was provided involving a third reviser for a final decision if necessary. Data extraction included general details corresponding to the characteristics of the studies (e.g., author, year of publication, sources of funding) and precise aspects about the type of enzymes and proteins used, the gingival condition of the population investigated, the time of administration, and follow-up.

### 2.4. Quality Assessment

The risk of bias was evaluated according to the Revised Cochrane risk-of-bias tool for randomized trials (RoB 2.0) [15]. This tool is structured into five domains of bias, focusing on different aspects of trial design, conduct, and reporting. Within each domain, a series of questions (‘signaling questions’) aim to elicit information about features of the trial that are relevant to risk of bias. A proposed judgment about the risk of bias arising from each domain is generated by an algorithm, based on answers to the signaling questions. The judgment can be ‘Low’ or ‘High’ risk of bias, or it can express ‘Some concerns’. Non-randomized studies were evaluated with the Cochrane risk-of-bias tool in Non-Randomized Studies (ROBINS-I) [16].

## 3. Results

### 3.1. Study Selection

**Figure 1:**
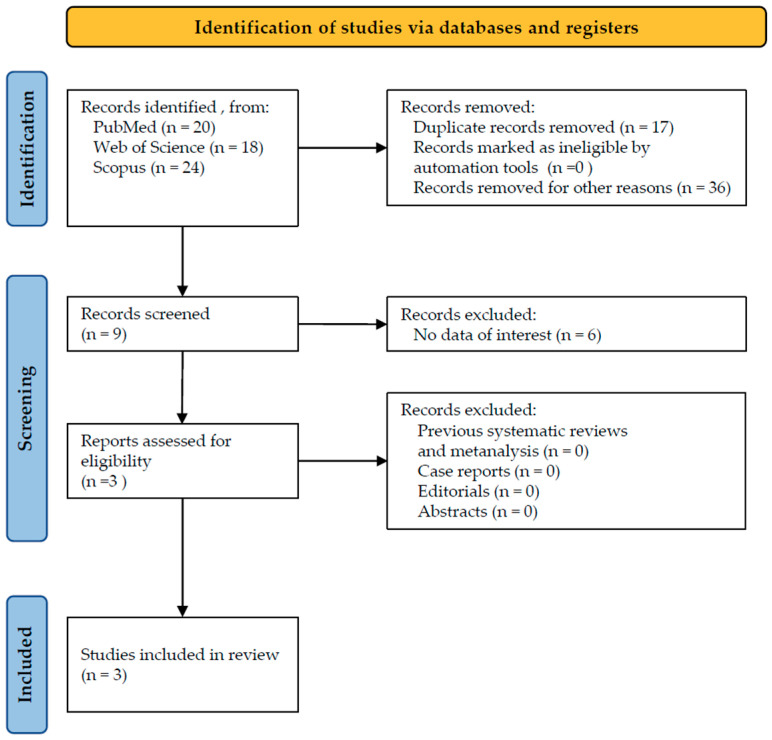
PRISMA flowchart.

The starting search supplied a total of 62 studies: 20 from PubMed, 18 from Web of Science, and 24 from Scopus. No studies were deleted due to being ineligible by automation tools, while 17 studies were removed because of duplication. Overall, 36 studies were removed for other reasons, for example, for analyzing other molecules, such as triclosan, stannous fluoride, and phytocannabinoids. A total of nine studies were selected for the screening phase, and a total of six studies were withdrawn because they failed to demonstrate any data of interest, for example, because they were conducted in vitro, in animals, on postbiotics, or on chemotherapy-induced oral mucositis. Eligibility was assigned to three studies based on their abstracts; no studies were erased for being systematic reviews, abstracts or editorials. For this reason, a total of three studies were incorporated into the inclusion phase (Figure 1) and analyzed according to their full text.

### 3.2. Detailed Results

Regarding the population age, 33% (1/3) of the studies referred to young adults (<25 y.o.), 33% (1/3) referred to adults (30–40 y.o.), and 33% enrolled people from all ages (18–56 y.o.). None of the studies selected investigated any gender differences. Regarding the periodontal indexes used, 66% (2/3) used the Modified Gingival Index (MGI), the gingival Bleeding Index (BI) and plaque assessments by use of the Modified Quigley and Hein index; 33% (1/3) used the Decayed Missing Filled Teeth (DMFT) index, the Gingival Bleeding Index (GBI), and the Plaque Control Record. Only one study involved a cut-off value for the MGI, in the range of 2.00–2.75. Just 33% (1/3) examined the effect of the tested toothpaste on black stains. All studies excluded subjects with systemic illness, periodontitis, and ongoing antibiotic intake in the last 3 months. The follow-up periods were heterogeneous: from 12 weeks and 14 weeks to one year.

### 3.3. Quality Assessment Results

**Figure 2:**
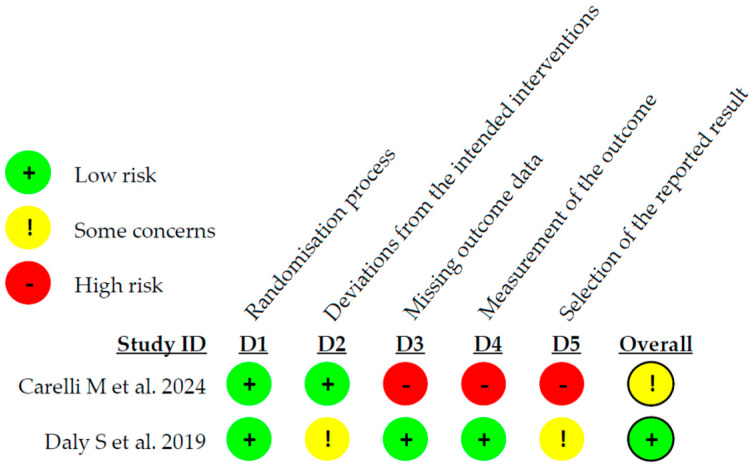
Risk of bias of the RCTs included in the systematic review [17,18].

**Figure 3:**
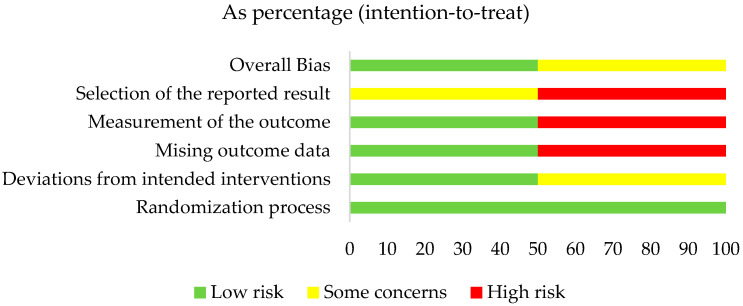
Overall risk of bias, with each category presented as percentages.

The risk of bias assessment of the two RCTs included was carried out using the Cochrane RoB 2.0 tool. The study by Carelli M. et al. [17] was classified as having some concerns regarding the reported results; the study by Daly S. et al. [18] had a low risk of bias. The details are presented in Figure 2. The overall risk of bias, with each category presented as percentages, is presented in Figure 3.

**Figure 4:**
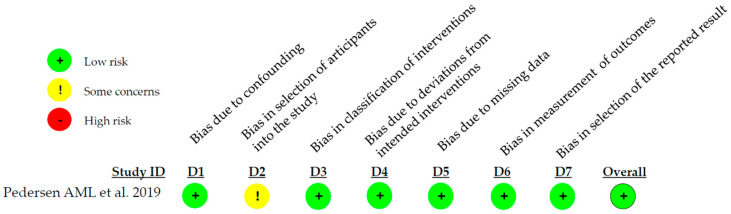
Risk of bias of the N-RCT included in the systematic review [19].

The risk of bias assessment of the included N-RCT was carried out using the Cochrane ROBINS I tool. The study by Pedersen AML. et al. [19] was classified as having a low risk of bias (Figure 4).

The totality of the included studies, including authors, year, population, age, main ingredients, purpose of administration and follow-up, and systemic issues of the investigated subjects, is presented in Table 1. Finally, a brief narrative summary of the included studies is displayed in Table 2.

## 4. Discussion

Oral health balance is shaped by the oral microbiome. This review aimed to investigate the impact of protein- and enzyme-containing toothpastes on individuals with gingivitis, focusing on clinical periodontal indexes as indicators of oral health status. A healthy oral microbiome maintains a dynamic equilibrium, where beneficial species predominate over harmful ones. This microbial diversity is crucial for preventing the overgrowth of single species, which can lead to diseases such as gingivitis [11].

The protective attributes of saliva are derived from a diverse array of components, including enzymes, proteins, and antimicrobial peptides [20]. The formation of dental plaque or biofilm is a key element in the pathomechanism of gingivitis and periodontitis.

The literature provides evidence that salivary LPO possesses the capacity to inhibit biofilm formation at various stages of its development [21]. Owing to its ability to adsorb onto the salivary pellicle [22], the LPO system has been demonstrated to effectively prevent the adhesion of precursor cariogenic microorganisms [23]. Comparable studies have been conducted on both single- and multispecies biofilms. Cawley et al. established that the LPO system, in conjunction with lactoferrin, lysozyme, and bovine milk immunoglobulins, can inhibit the formation of single-species *S. mutans* biofilms. This discrepancy can be attributed to the LPO system’s ability to kill microorganisms while being unable to inactivate glucosyltransferases, the extracellular bacterial enzymes responsible for synthesizing glucans, the structural components of the biofilm matrix [24].

Additionally, by disrupting the bonds within peptidoglycan, lysozyme induces bacterial lysis, leading to the rupture of bacterial cells and the inhibition of their metabolic activities [25]. Lactoferrin, a glycoprotein present in saliva with an iron-binding affinity, sequesters free iron within the oral environment, thereby depriving bacteria of this essential nutrient and restricting their growth. It demonstrates direct bactericidal activity against specific cariogenic and periodontal pathogens [26].

Another critical component of the salivary defense system is represented by sIgA, which binds to bacteria, thereby preventing their adherence to oral surfaces and neutralizing bacterial toxins [27]. Due to the importance of saliva in the complex oral ecosystem, dentifrices with natural enzymes and proteins were developed. The enzymes typically contained in these products are glucose oxidase, lactoperoxidase, and amyloglucosidase, as well as proteins like lactoferrin and lysozyme, mirroring the enzyme and protein composition found in saliva [28].

Regarding the studies included in the present review, Carelli et al. [17] in a 14-week RCT found that using a toothpaste containing enzymes and salivary proteins did not reveal significant alterations in the oral microbiota or oral health of test subjects compared to controls. While a reduction in certain pathogenic bacteria (*P. gingivalis*, *P. intermedia*, *A. actinomycetemcomitans*, *A. naeslundii*, *T. forsythia*, *T. denticola*, *Actinomyces* spp., *Veillonella* spp.) was observed in the saliva of test subjects, no changes were noted in dental plaque. These findings were interpreted by the authors as the test toothpaste possibly having limited efficacy against planktonic bacteria in saliva but little or no impact on the bacteria that form dental plaque biofilm. However, the study was limited to a small sample of 16 patients with black stains followed for a short time.

The RCT by Daly S. et al. [18] indicates that brushing with a toothpaste containing natural proteins and enzymes is more effective in preventing gingivitis than a commercially available fluoride toothpaste with strong anti-plaque properties and does so without any adverse side effects. Even if the sample size was adequate, with 229 participants, in this case, the follow-up period was 13 weeks.

Finally, the N-RCT of Pedersen AML et al. [19] stated that the consistent use of a fluoride toothpaste containing enzymes and proteins for at least a year is linked to better gum health than other fluoride toothpastes without these additional ingredients. Nevertheless, the current study and the previous one exclusively included participants with good oral health and no need for treatment for periodontitis or dental caries. Consequently, the findings may not be applicable to individuals with overt oral diseases like periodontitis or dental caries. Additionally, none of the studies included collected data on socioeconomic status, a known factor influencing oral health [29] and the composition of the oral microbiota [30]. Based on the reviewed studies, toothpastes containing enzymes and salivary proteins show promise in improving oral health. However, the evidence is limited by factors such as small sample sizes, short follow-up periods, and a focus on participants with good oral health.

The toothpaste tested in the studies included in this review contains fluoride and the mild surfactant Steareth-30 instead of sodium lauryl sulfate (SLS). In recent in vitro and in vivo studies, Steareth-30 caused minimal gingival sloughing as compared to SLS [31]. However, patients were not directly asked to rate their experience following the use of this toothpaste in terms of satisfaction with the taste or any oral discomfort; this is a limitation of all the studies included that should be addressed in future clinical studies.

Overall, while the evidence suggests that toothpastes containing enzymes and salivary proteins can be beneficial for oral health, further research is needed to fully understand their efficacy, particularly in individuals with existing oral diseases.

The oral cavity is equipped with several defense mechanisms, including the continuous flow of saliva—which acts as a physical barrier and contains antimicrobial proteins such as lysozyme and lactoferrin [32]—and the epithelial integrity of the oral mucosa. Additionally, the oral microbiome itself contributes to immune defense by outcompeting pathogenic microbes for available niches and nutrients [33]. Toothpaste fluoride strengthens tooth enamel by promoting remineralization and reducing enamel demineralization, thus mitigating the risk of dental caries [34]. Other components, such as xylitol and chlorhexidine, exhibit antibacterial properties that can modify the oral microbiota, potentially reducing the abundance of harmful bacteria and promoting a more balanced microbial ecosystem [35,36]. Finally, natural salivary lactoferrin and lysozyme show a potential role in enhancing the innate oral defense [37,38]. These interactions between toothpaste ingredients and oral protection mechanisms underscore the importance of oral care products in maintaining local eubiosis. In an observational clinical trial by Berlutti F. et al. [39] involving the topical administration of bovine lactoferrin (bLf) to volunteers with periodontitis, bLf was shown to reduce cytokine levels, including IL-6 in crevicular fluid, as well as edema, bleeding, pocket depth, and both gingival and plaque indexes, leading to improved clinical attachment levels. While further clinical trials are needed, these findings provide compelling evidence supporting the therapeutic potential of this multifunctional natural protein. Additionally, lysozyme has been shown to modulate inflammation and interact synergistically with other salivary components, such as lactoferrin, to enhance the overall antimicrobial and anti-inflammatory responses. Through these mechanisms, lysozyme helps maintain oral health and supports the preservation of oral homeostasis by preventing microbial dysbiosis [40].

### 4.1. Limitations

One of the challenges encountered in our analysis was the variability in the periodontal indexes used across the studies, making it difficult to directly compare and draw definitive conclusions. The presence of heterogeneous samples, different ages, different kinds of clinical measurements, and different follow-up periods were all confounding factors. Additionally, the diversity in the inclusion criteria for participants in the included studies represents a severe bias and makes comparison between studies unclear. The scarce number of studies incorporated in this review reflects a notable deficiency in the current body of research.

### 4.2. Clinical Relevance

The present systematic review highlighted that a toothpaste with an enzymatic technology (amyloglucosidase, glucose oxidase, and lactoperoxidase) and salivary proteins (lysozyme and lactoferrin) could be useful and effective in preventing gingivitis and in managing gingival inflammation, both in the short and long term, thanks to the proved reduction in the Modified Gingival Index (MGI), Bleeding Index (BI), Plaque Index (PI). The authors acknowledge the limited number of eligible studies included in this systematic review. This scarcity indeed reflects a current gap in the literature, which underscores the importance of our work. By systematically identifying and analyzing the available evidence—even if limited—the authors aim to highlight the need for further research and draw attention to an understudied but potentially significant area.

## 5. Conclusions

The available evidence on the use of toothpastes with enzymes and proteins and on their effect on gum health is poor. Only three studies were eligible for the purposes of the present review, and one of them presented some concerns for the quality assessment. The other two studies shared the idea that a toothpaste based on enzymes and proteins could be more effective in plaque and gingival inflammation control than a standard fluoride dentifrice. Despite these limitations, the positive findings regarding gum health improvement suggest that a toothpaste containing enzymes and proteins could be a valuable addition to oral care regimens. Moreover, future well-designed clinical studies are needed to assess the behavior of gingival indexes and microbiological changes in people using a toothpaste with enzymes and proteins.

## Figures and Tables

**Table 1:** Main results. RCT, randomized controlled trial. N-RCT, non-randomized clinical trial. DMFT, Decayed Missing Filled Teeth. GBI, Gingival Bleeding Index. MGI, Modified Gingival Index. BI, Bleeding Index. PI, Plaque Index. Age is expressed by mean value or range.

Authors/Year	Study Design	Population /Age	Enzymes/ Proteins	Aim of Administration	Follow-Up	Systemic Conditions
Carelli M. et al., 2024 [17]	RCT	26/22.5	Amyloglucosidase, glucose oxidase, lactoperoxidase, lysozyme, lactoferrin, bovine colostrum (IgG)	Effects on DMFT, gingival indexes (GBI, plaque control) and black stains	14 weeks	Healthy
Daly S. et al., 2019 [18]	RCT	229/32.6	Amyloglucosidase, glucose oxidase, lactoperoxidase, lysozyme, lactoferrin, bovine colostrum (IgG)	Effects on gingival indexes (MGI, BI, PI modified by Quigley and Hein)	12 weeks	Healthy
Pedersen AML. et al., 2019 [19]	N-RCT	305/18→56	Amyloglucosidase, glucose oxidase, lactoperoxidase, lysozyme, lactoferrin, bovine colostrum (IgG)	Effects on gingival indexes (MGI, BI, PI modified by Quigley and Hein)	12 months	Healthy

**Table 2:** Summary of the included studies.

Authors/Year	Conclusions
Carelli M. et al., 2024 [17]	Brushing with an electric toothbrush seemed to be more effective in reducing black stains compared to a manual brush. This improvement was seen regardless of the toothpaste used.
Daly S. et al., 2019 [18]	Brushing with the enzyme and protein toothpaste resulted in lower plaque and bleeding indexes.
Pedersen AML. et al., 2019 [19]	Using a fluoride toothpaste containing enzymes and proteins for at least 1 year is associated with improved gum health compared to other fluoride toothpastes lacking these additional ingredients.

## Data Availability

No new data were created or analyzed in this study.
